# Laparoscopic Assisted Percutaneous Anterior Gastropexy for the Management of Acute and Chronic Gastric Volvulus in Infants

**DOI:** 10.3390/children9091275

**Published:** 2022-08-24

**Authors:** Roberta Valentina Iacona, Francesco Grasso, Silvia Antonia Grimaldi, Massimo Lebet, Sebastiano Cacciaguerra

**Affiliations:** Department of Pediatric Surgery, Azienda Ospedaliera di Rilievo e di Alta Specializzazione Garibaldi—ARNAS-Nesima, 95024 Catania, Italy

**Keywords:** gastric volvulus neonates and infants, anterior gastropexy infants, laparoscopic gastropexy neonates and infants

## Abstract

Acute and chronic gastric volvulus (ACGV) is a rare event in infants and children. Its prompt treatment is needed to avoid gastric ischemia and perforation. A laparotomy or a laparoscopic/endoscopic reduction with or without the gastrostomy formation has been described to treat this condition. We wanted to report our experience and describe the surgical technique used to perform the percutaneous laparoscopic assisted anterior gastropexy in neonates presenting with this condition. We perform a retrospective review of a single institution’s experience with laparoscopic assisted percutaneous anterior gastropexy over a seven-year period (2015–2022). Procedures were performed under general anesthesia and the anterior gastropexy was performed using a modified extracorporeal knotting technique as described for the laparoscopic assisted repair of inguinal hernias via percutaneous internal ring suturing. Thirteen patients underwent surgery for ACGV at our institution over a seven-year period. The median age at diagnosis was 57 days, 7/13 patients presented with acute vomiting and regurgitation (54%), 1/13 (8%) presented with mainly feeding difficulties and 1/13 (8%) presented with acute abdominal distension. Data were not available for 4/13 patients. All of the patients underwent laparoscopic assisted anterior gastropexy using extracorporeal knotting technique; no gastrostomy insertion was needed. The median operative time was 50 min (40–95 min). No intraoperative complications were reported. Post-operatively patients were started on feeds on day 3 (2–5 days). Only one patient (8%) developed a postoperative complication: subcutaneous granuloma at the extracorporeal knot site. Although rare, acute GV is an important cause of gastric outlet obstruction with a detrimental outcome if not promptly recognized and treated. Laparoscopic assisted percutaneous anterior gastropexy is an attractive and safe alternative for the management of this condition in both infants and older children. This technique does not require gastrostomy placement and it has a very low morbidity rate with no mortality reported.

## 1. Introduction

First described in 1866 by Berti, gastric volvulus (GV) in infants and children is a rare pathological entity, with an estimated incidence not clearly reported in literature often diagnosed with delays as its presenting symptoms are similar to more common conditions such as pyloric stenosis and gastroesophageal reflux [1,2,3,4].

According to the gastric axial rotation, GV is classified as organo-axial, mesenterico-axial and combined or mixed [5]. Equally, depending on its presentation symptoms and causes, GV can also be classified as acute or chronic and primary or secondary. Lastly, depending on the site, GV can be intrathoracic or intra-abdominal [2].

GV occurs when the stomach rotates more than 180° around an axis, leading to gastric outlet obstruction. Anatomical anomalies have been considered responsible for GV, such as the absence or loss of gastrocolic and gastrosplenic ligaments [5].

Because of the risk of gastric ischemia and perforation—secondary to stomach twisting—and an estimated mortality rate of 6.4% [6], its prompt correction is mandatory and, in fact, several surgical techniques have been proposed and used over the years.

Here we report our institution’s experience with laparoscopic assisted percutaneous anterior gastropexy for the treatment of ACGV in neonates and infants, using the extracorporeal knotting technique first described in 2006 for the repair of indirect inguinal hernia [7].

## 2. Material and Methods

We performed a retrospective review of clinical and operative notes of patients presenting to a single Tertiary Center for Pediatric Surgery—ARNAS Garibaldi, Catania, Sicily, Italy—between January 2015 and March 2022, with ACGV. Data on demographics, comorbidities, clinical presentation, symptoms and their duration were reviewed. Operative notes were examined, and the surgical approach used was described including patient decubitus, as well as the instruments used and operative time. Descriptive data analysis was performed. Any intra- and post-operative complications were reviewed. The revision of the published literature on the management of acute and chronic gastric volvulus was performed via Pubmed, Embase, Medline, Google Scholar and Cochrane database.

## 3. Results

### 3.1. Demographics

From January 2015 until March 2022, 13 patients presented to our Emergency Department with symptoms of gastric outlet obstruction requiring hospital admission and further investigations. 

Our center is a referral tertiary hospital for pediatric surgery, covering an estimated population of 1.5 million habitants from six different cities (Catania, Siracusa, Enna, Agrigento, Ragusa and Caltanissetta).

Overall, 7/13 (54%) were female, median age at surgery was 57 days (6–122 days), median weight—4840 g (3220–6000 g). Data on comorbidities were available for 9/13 patients: 4/9 (44%) presented associated anomalies (PFO, PDA, ileal duplication); all of the comorbidities were known prior to the surgery except for the ileal duplication, which was diagnosed intraoperatively and addressed separately. Seven (54%) patients were admitted with vomiting and regurgitation, 1/13 (8%) presented with feeding difficulties and failure to thrive and 1/13 (8%) presented with sudden abdominal distension. Data on clinical presentation on admission were not available for 4/13 patients. Overall, the duration of symptoms varied from 24 h (sudden abdominal distension) to 2 weeks (feeding difficulties, failure to thrive) [Table 1].

**Figure 1:**
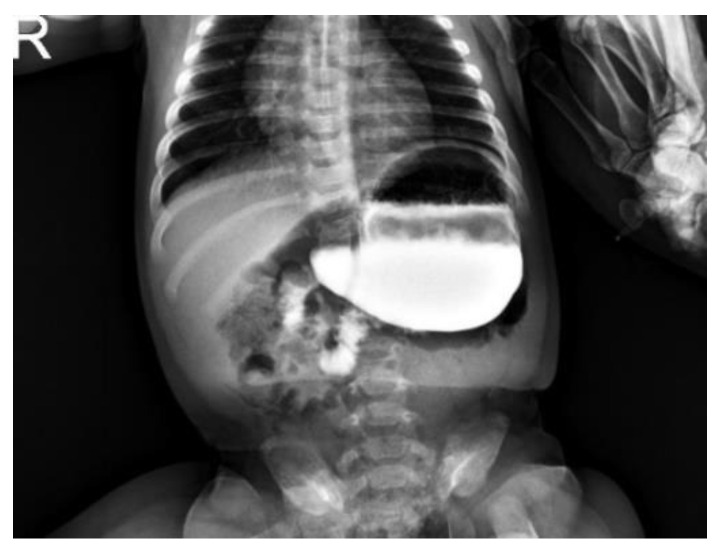
Upper GI contrast study: 4 months old infants presenting with vomiting and failure to thrive. The contrast report showed an enlarged stomach, the gastric bubble projecting above the cardias, a more elevated left hemidiaphragm and the gastric antrum higher than the pylorus as for organo-axial volvulus.

**Figure 2:**
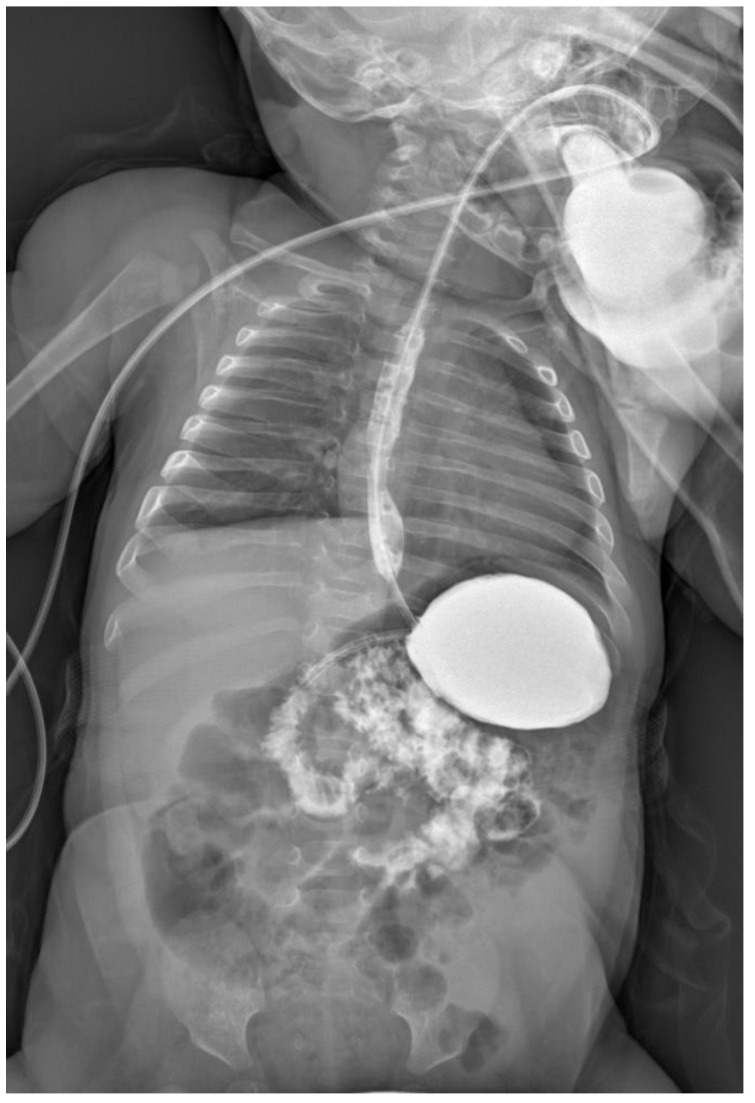
Upper GI contrast study: 6 months old girl presenting with ongoing vomiting and failure to thrive. The contrast study shows an enlarged stomach horizontally orientated, the antrum cranially displaced to the pylorus as per organo-axial gastric volvulus.

All of the patients received adequate resuscitation with endovenous administration of normal saline 0.9% and glucose 5% and NG tube. Nine (70%) underwent US abdomen to exclude pyloric stenosis as cause of gastric outlet obstruction. No data were available for 4/13 patients in regard to the US findings. All of the patients underwent an upper GI contrast study showing enlarged and rotated stomach with delayed or absent emptying and pylorus looking downward, suggestive and diagnostic of organo-axial volvulus [Figure 1 and Figure 2].

### 3.2. Operative Technique

**Figure 3:**
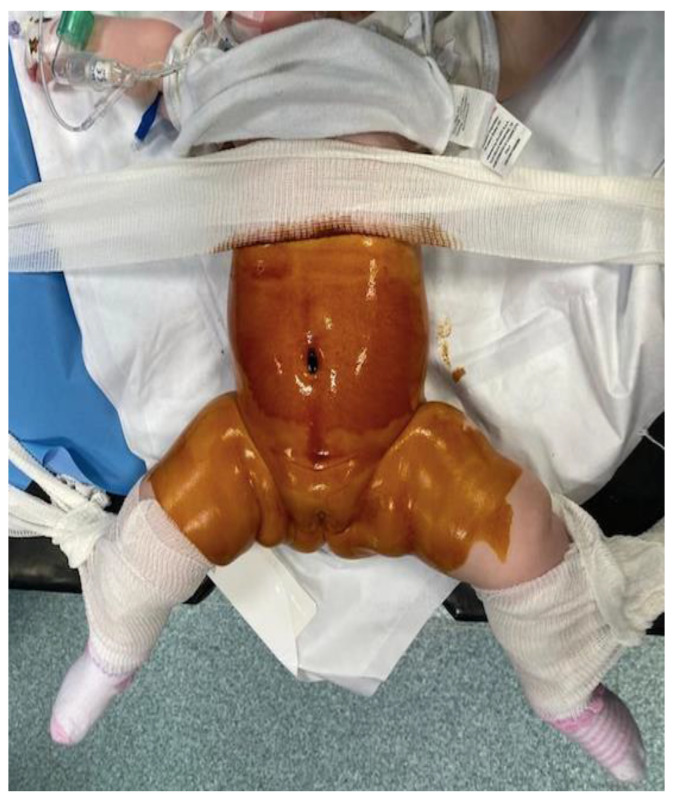
Intraoperative image: Patient decubitus.

A standard supine position was used for all of the patients [Figure 3]. Patients were positioned at the end of the bed with the legs in frog position properly secured. We used a 5 mm infra-umbilical port and a 5 mm 30° scope. Pneumoperitoneum was established with an intra-abdominal pressure of 8–10 mmHg and 1–1.5 lt/min was used. A stab incision within the right upper quadrant was performed to accept a 3 mm instrument inserted under direct vision, without using a trocar.

**Figure 4:**
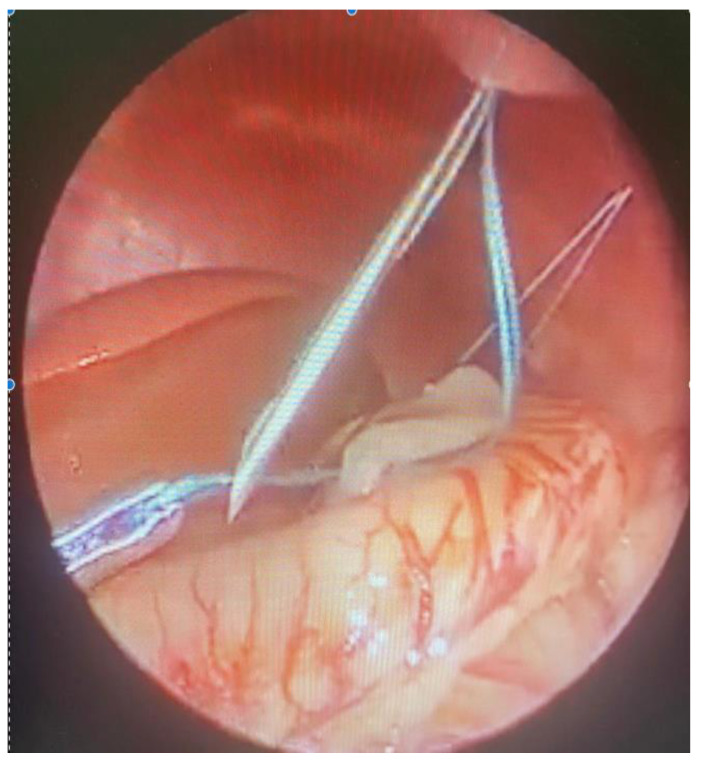
Intraoperative image: intracorporeal. Suture insertion via 14 G needle.

**Figure 5:**
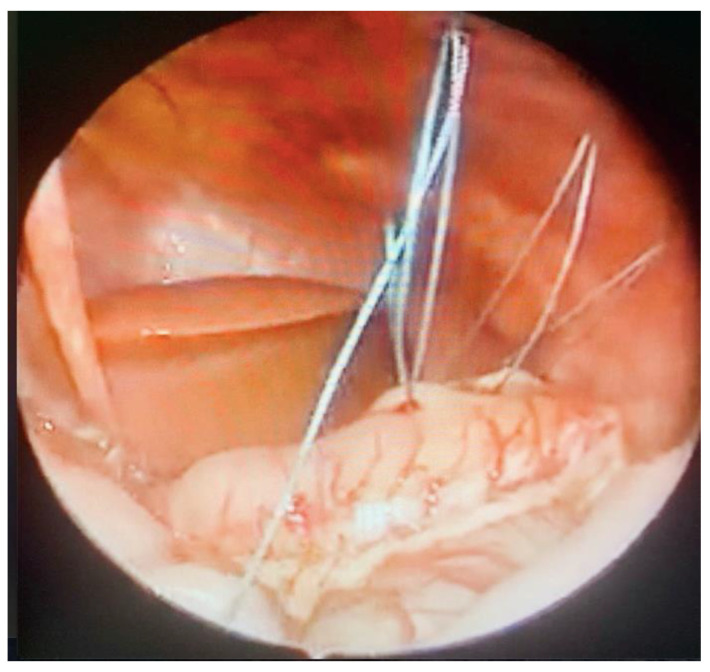
Intraoperative image: positioning of seromuscular thread within the stomach’s greater curvature and the anterior abdominal muscle wall.

**Figure 6:**
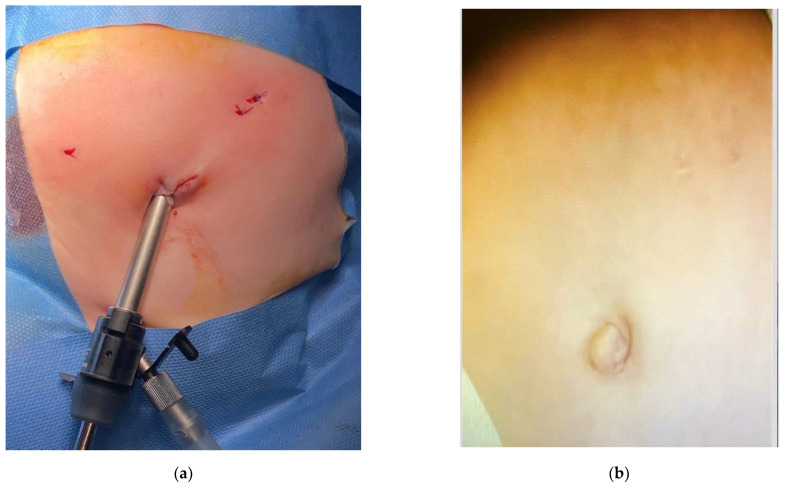
(**a**) Intraoperative image: end of the procedure, aspect of the extracorporeal suturing. (**b**) same patient attending follow-up clinic, cosmetic result.

Under direct vision, a 14 Gauge injection needle containing a non-absorbable suture (2-0 monofilament thread non reabsorbable) was inserted through the abdominal wall within the abdominal cavity and a seromuscular bite was passed through the anterior wall of the stomach. The needle was pulled out leaving the loop inside the seromuscular aspect of the stomach. Another thread end was then introduced again into the barrel of the needle and the needle passed through the same skin puncture point, the end of the thread goes through the barrel of the needle into the loop of the thread loop and the needle was then withdrawn. The knot was tied to secure the anterior gastric wall to the abdominal wall under direct vision Figure 4, Figure 5 and Figure 6]. The procedure is repeated twice on the anterior gastric wall within the greater curvature. We did not position gastrostomies and did not perform anti-reflux procedures.

During the laparoscopic exploration, we did not find diaphragmatic defects such as diaphragmatic hernias or eventration; none of the patients was found to have signs of gastric ischemia intraoperatively. In fact, only one patient (7.6%) was found to have a gastric volvulus intraoperatively requiring de-rotation. No information was found for 3/13 patients in regard to the presence of volvulus intraoperatively. In all of the cases, the stomach was described as very enlarged and floppy with ligamentous laxity.

The umbilical access was closed with 2-0 or 3-0 absorbable braided suture; the skin was closed with 4-0 absorbable braided material. The median operative time was 50 min (40–95 min). All of the patients received antibiotic prophylaxis with Amoxicillin and Clavulanate or Ampicillin.

### 3.3. Outcomes

No intraoperative complications were reported. Post-operatively patients were fed on day 3 (min 2–max 5 days), the main delay was related to the patient being found to have an ileal duplication causing rectal bleeding and requiring a second surgical procedure. All of the patients were discharged on anti-reflux medications (PPI) which were stopped by the first post-operative follow-up.

Post-operatively one patient (8%) developed a subcutaneous granuloma at the extracorporeal knotting site. The follow-up period ranged from four months to seven years: to date, all of the 13 patients report not having experienced recurrence of symptoms and none required revision surgery or a planned anti-reflux procedure.

## 4. Discussion

The stomach is a hollow viscus normally fixed within the abdominal cavity by the presence of four ligaments: gastrocolic, gastrohepatic, gastrophrenic and gastrosplenic; moreover, the pylorus and the gastroesophageal junction keep the stomach in place, avoiding its twisting and therefore its volvulus. When these ligaments are lacking or loose, the stomach can move freely along its axis and twist. If this happens on its longitudinal axis passing between the esophagogastric junction and the pylorus, an “organoaxial volvulus” will occur; if instead the twist happens on its perpendicular axis—as if an imaginary line passes from the lesser to the greater curvature—a “mesenterico-axialis/mesentero-axial volvulus will happen. Rarely the stomach can rotate in both ways leading to a “combined volvulus” [2,8] Usually GV can occur acutely (43%) or chronically; generally, males are more commonly affected (54%) and usual pathological associations include: congenital diaphragmatic hernia (17%), diaphragmatic eventration (25%), malrotation (7%), wandering spleen (6%), asplenia (6%) [9]. In terms of age at presentation and type of volvulus, the published literature states that 21% of children with acute GV present within their first month of life and that 54% will usually have an organo-axial volvulus [10]. Our cohort has a median age of 57 days–2 months—and only one (8%) patient presented with acute gastric volvulus confirmed intraoperatively. Interestingly, the only one patient presenting with acute volvulus was 6 days old confirming the literature data and suggesting that chronic/intermittent volvulus manifestation will be subtler. Equally, all of our patients had a radiological or intraoperative diagnosis of organo-axial volvulus confirming this type as the most common type of gastric volvulus in children and infants. In terms of clinical presentation, in 1904 Borchardt described the clinical triad of the presentation of GV as inability to vomit, severe epigastric distension and inability to pass a nasogastric tube [8]. Usually, the most common symptoms are non-bilious vomiting, epigastric distension and abdominal pain. Fifty-four percent of our patients presented with persistent vomiting and failure to thrive, 8% presented acutely with epigastric distension whilst none of our patients presented with the classic Borchardt triad. Whilst the mortality rate reported in the literature is 7%, we did not experience mortality [8,9]. All of our patients underwent an upper GI contrast study, suggestive of an organoaxial volvulus, which was diagnostic for gastric volvulus. As reported in literature, classic radiological signs of GV are: (1) a pulled up greater curvature of the stomach, (2) a horizontal gastric shadow, (3) a hairpin sign, (4) minimal gas in the small and large bowel, (5) a nasogastric tube trapped in the lower end of the esophagus, (6) the greater curvature crossing the esophagus [11]. In our experience, we observed the stomach being enlarged with a cranial displacement of the antrum above the pylorus level and the elevation of the hemidiaphragm. In our experience, patients presented with both acute and chronic issues; the treatment of gastric volvulus can vary depending on its presentation—while acute GV undoubtedly requires emergency treatment, the management of chronic gastric volvulus remains debated. In 2007, Al-Salem [2] suggested that chronic GV should be treated conservatively by modifying the child’s decubitus position and keeping them prone with a slight head up, reducing the amount of air and gastric contents regurgitation into the esophagus. The same author also suggested the use of medications such as metoclopramide to enhance esophageal and gastric emptying and H2 blockers to prevent esophageal ulceration. In his report, this management was reported as effective in treating the condition [2]. In our experience, we found that our patients presenting with chronic gastric volvulus were experiencing severe symptoms such as failure to thrive and inability to feed properly leading to a progressive deterioration of their clinical conditions. As a result, we feel that surgical treatment should be offered to these patients as chronic gastric volvulus is probably associated with spontaneous resolution until it becomes acute and irreversible [11,12].

As mentioned, over the years many surgical approaches have been described in the literature to manage ACGV: laparotomy and gastropexy (with or without gastrostomy), laparoscopic surgery and endoscopic reduction [10]. An interesting paper was published in 2006 by Patkowski et al. [7,13,14] describing their novel technique for the repair of inguinal hernias in children called the PIRS technique and using a laparoscopic-assisted percutaneous knotting technique. Since then, this technique has gained popularity and is widely used. We thought to apply the benefit of the extracorporeal knotting technique, (avoiding the difficulties of training in intracorporeal knotting and increasing the timing of the surgical procedure) the avoidance of unnecessary trocars and gastrostomy insertion to be applied to patients presenting with ACGV. We applied the extracorporeal knotting technique as a possible technical variation to perform the anterior gastropexy. In our opinion this surgical technique has the advantage of being minimally invasive and it also avoids the need for gastrostomy placement, sensibly reducing morbidity and improving patient’s symptoms as we experienced with our patients at the follow-up.

## 5. Conclusions

ACGV is a rare condition that should be taken into account in pediatric patients presenting with non-bilious vomiting, abdominal distension, feeding difficulties and failure to thrive. Radiological findings are important to warrant promptly the correct diagnosis and quickly establish the treatment. While surgery seems to be the correct approach for the management of acute gastric volvulus, whether chronic gastric volvulus should be treated at all is still debated. We feel that surgical treatment should be reserved for patients presenting with progressive deterioration of their clinical conditions and we feel that the minimally invasive approach described is a safe and adequate option avoiding gastrostomy placement and with minimal morbidity. A wider cohort and a prospective study would be needed to establish the best option for this condition.

## Figures and Tables

**Table 1:** Patient demographics and presenting symptoms.

Gender	Gestational Age	Age at Surgery (Days)	Weight at Surgery	Presenting Symptoms	Type of Volvulus
F	38 + 5	102	5650	Vomiting and retching	Organo-axial
F	39 + 0	122	4840	Vomiting and retching	Organo-axial
F	38 + 5	57	4600	Abdominal distension	Organo-axial
M	39 + 1	36	3220	Vomiting and retching	Organo-axial
F	39 + 2	6	3680	Vomiting and retching	Organo-axial
M	38 + 2	57	5280	Vomiting and retching	Organo-axial
M	38 + 0	78	6000	Vomiting and retching	Organo-axial
M	39 + 0	43	3890	Vomiting and retching	Organo-axial
F	N/A	N/A	N/A	N/A	Organo-axial
M	40 + 4	69	4980	Feeding difficulties/failure to thrive	Organo-axial
F	N/A	N/A	N/A	N/A	Organo-axial
M	N/A	N/A	N/A	N/A	Organo-axial
F	N/A	180	N/A	N/A	Organo-axial

## Data Availability

Not applicable.
